# Thin Film Composite and/or Thin Film Nanocomposite Hollow Fiber Membrane for Water Treatment, Pervaporation, and Gas/Vapor Separation

**DOI:** 10.3390/polym10101051

**Published:** 2018-09-20

**Authors:** Kailash Chandra Khulbe, Takeshi Matsuura

**Affiliations:** Industrial Membrane Research Laboratory (IMRL), Chemical and Biological Engineering Department, University of Ottawa, Ottawa, ON K1N 6N5, Canada; matsuura@eng.uottawa.ca

**Keywords:** thin composite hollow fiber, water treatment, water vapor separation, dehydration of alcohols, pervaporation

## Abstract

Thin film composite (TFC) polymeric hollow fiber (HF) membranes are widely used in industrial gas/vapor separations and water treatment. There are many advantages of TFC HF membranes, such as low energy requirements, simplicity of operation, and high specificity. In the present article, a review is made on the progress that has been achieved during the past 15 years in the preparation of the HF substrate and the preparation/modification of the thin selective layer. The review also includes their applications in water treatment, dehydration of alcohols via pervaporation, and gas/vapor separation.

## 1. Introduction

Hollow fiber membranes (HFMs) are artificial membranes, with a semi-permeable barrier in the form of hollow fiber, were developed in the 1960s for reverse osmosis (RO) applications. Since then, they have been successfully applied in water treatment, desalination, cell culture, and in medical and pharmaceutical fields [1]. Hollow fiber membranes are highly efficient and very popular due to their low energy requirements, simplicity of operation, and high specificity qualities. The performance of an optimized hollow fiber nanofiltration (NF) module is 100% better than an optimized spiral-wound module [2]. A very large membrane area of HF can be packed into a single module, which is the most significant advantage.

A highly porous substrate coated with a dense film of a different polymer is usually called a thin film composite (TFC) membrane. For RO, a thin film composite (TFC) membrane is a multi-layer membrane in which an ultrathin semipermeable membrane layer is deposited on a finely microporous support structure [3]. Among several methods devised for the preparation of composite membranes, interfacial polymerization is used most often. Cadotte [4] fabricated for the first time the selective polyamide film by interfacial polymerization of m-phenylene diamine (MPD) and trimesoyl chloride (TMC), which is still used widely.

## 2. Method of Making Thin Film Composite (TFC) Hollow Fiber (HF)

### 2.1. Fabricating Hollow Fiber

There are four techniques to fabricate HFMs.

i Melt spinning: In this process, a melted thermoplastic polymer is extruded through a spinneret into air and is subsequently cooled.
ii Dry spinning: Polymer solution in an appropriate solvent is extruded through a spinneret into air.
iii Dry-jet wet spinning: Polymer solution in an appropriate solvent is extruded through a spinneret into air and a subsequent coagulant (usually water).
iv Wet spinning: Polymer solution in an appropriate solvent is extruded directly into a coagulant (usually water).

**Figure 1:**
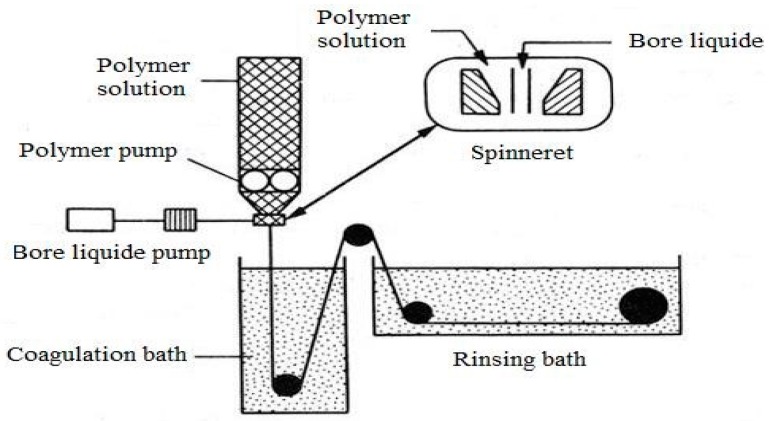
Apparatus for the fabrication of hollow fibers (HFs) [5].

A spinneret is used in all the above techniques. The use of a spinneret is common. A spinning dope, prepared by dissolving polymer in a proper solvent or by melting, is forced through a metal nozzle that has fine holes to form a filament. As soon as the dope solution emerges from the nozzle in the form of long fibers, it solidifies by evaporation of solvent, cooling, or coagulation. A schematic diagram for the formation of a hollow fiber is shown in Figure 1. The properties, such as average pore diameter and wall thickness of the HF, depend on the spinning parameters which are as follows:

i Dimensions of the spinneret;
ii driving force;
iii temperature;
iv length of air gap (for dry-jet wet spinning);
v coagulant;
vi composition of “dope” (polymer) and “bore” (coagulant) solutions; and
vii speed at which produced fiber is collected by a motorized spool.

During solidification following stages are involved:

i The diffusion of internal precipitant molecules into the concentrated hollow fiber forming polymer solution. At the same time, polymer coagulates (precipitates from solution) to form a solid phase;
ii in this stage, a cross-diffusion of solvent molecules from the polymer solution into the internal nonsolvent starts and it speeds up the coagulation of the polymer and the formation of the porous structure of the fiber body; and
iii during the evaporation of solvent molecules from the outer surface of the fiber into the air, the concentration of polymer on the outer surface of the fiber is increased, which is the cause of the formation of the dense (selective) polymer layer.

**Figure 2:**
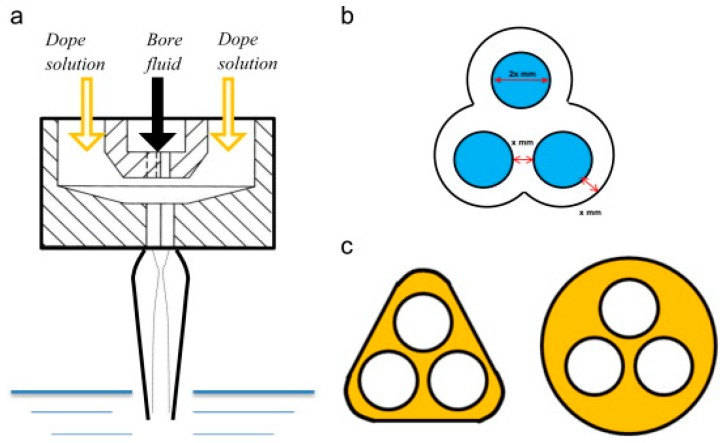
(**a**) Single layer tri-needle spinneret; (**b**) bottom view of the tri-needle spinneret; and (**c**) cross section [6].

Chung’s group [6] designed and fabricated a tri-bore blossom spinneret (Figure 2) for making a tri-bore thin-film composite (TFC) hollow fiber (HF) membrane.

### 2.2. TFC Hollow Fiber Fabrication

A composite membrane consists of a nano-thin selective dense layer and a porous substrate. By modifying the top selective layer and bottom porous substrate, the overall membrane performance can be maximised. There are many ways or techniques to make thin composite layers on the surface of a support. The most common ones are:

i Interfacial polymerisation reaction;
ii dipping method;
iii plasma treatment; and
iv chemical reaction.

There are also three other methods [7], but these are not very common:

i Chemical vapour deposition (CVD);
ii sputtering; and
iii spray pyrolysis.

The spray pyrolysis method has been successfully applied to produce fine metals or metal oxide particles. Li et al. [8] obtained a Pd-Ag alloy membrane on the surface of a porous alumina hollow fiber by spray pyrolysis of Pd(NO_3_)_2_ and AgNO_3_ solutions on a H_2_-O_2_ flame.

#### 2.2.1. Dip Coating

In industries, coated fabrics are manufactured by dip coating. This technique is also common in academic research where thin film coating is needed. Thin film composite hollow fiber membranes are fabricated in the same way. There are five stages in the dip coating process [9]:

i Immersion: With a constant speed, the substrate is immersed in the solution of the coating material (preferably jitter-free);
ii start-up: The substrate has remained inside the solution for a while and is starting to be pulled up;
iii deposition: The thin layer deposits itself on the substrate while it is pulled up. To avoid jitters, the withdrawing should be at a constant speed. The thickness of the coating depends on the withdrawal speed (faster withdrawal gives a thicker coating material) [10,11];
iv drainage: Excess liquid will drain from the surface; and
v evaporation: A thin layer is formed on the surface by evaporation of the solvent. For volatile solvents, such as alcohols, evaporation starts during the deposition and drainage steps.

To fabricate thin films by self-assembly via the sol-gel technique, the dip coating method is generally used. Self-assembly can give a film thicknesses of exactly one monolayer. The sol-gel technique creates films of an increased, precisely controlled thickness that are mainly determined by the deposition speed and solution viscosity. Functional coatings are applied to change the surface properties of the substrate, such as adhesiveness and wettability.

#### 2.2.2. In-Situ Polymerization

**Figure 3:**
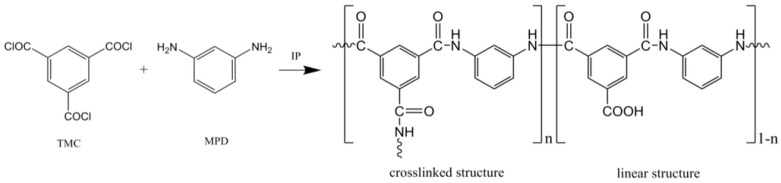
Interfacial polymerization (IP) between trimosyl trichloride (TMC) and 1,3-phenylenediamine (MPD).

The most common technique to make a TFC hollow fiber membrane is by interfacial polymerization (IP) reaction. IP reaction to form polyamide dense layer is based on trimosyl trichloride (TMC) and 1,3-phenylenediamine (MPD). Figure 3 shows the IP between TMC and MPD. During IP, polymerization occurs at the interface between an aqueous solution containing one monomer and an organic solution containing a second monomer. The interfacial polymerization is a self growth polymerization. IP occurs in a mixed monolayer of the adsorbed monomers. When the interfacial pressure of the adsorbed mixed monolayer exceeds the equilibrium spreading pressure of the polymer, the latter is precipitated from the monolayer, giving rise to a thick film at the interface. There are numerous unstable oligomers (molecules) that must be synthesized in situ (i.e., in the reaction mixture, but cannot be isolated on their own) for use in various processes. Mostly, in TFC HF fabrication, the selective layer is deposited on the lumen side of the fiber.

Urper et al. [12] discussed the recent developments in the design of TFC hollow fiber nanofiltration membranes and provided a comparative analysis of the two main methods of their fabrication:

i Interfacial polymerization; and
ii phase inversion.

To prepare integrally skinned hollow NF, the direct phase inversion (PI) method is commonly used in which the porous sublayer and the separation layer (membrane skin) are made of the same polymer. On the other hand, IP on phase-inverted membranes is used to prepare thin film composite (TFC) membranes where the porous support is made of a different material (typically polysulfone) than the skin layer (typically aromatic polyamide).

## 3. Water Treatment

Different types of membranes are used for softening, disinfection, organic removal, and desalination of water and wastewater. There are four types of membranes that are used for water treatment:

i Ultrafiltration (UF);
ii reverse osmosis (RO);
iii nanofiltration (NF); and
iv microfiltration (MF).

**Figure 4:**
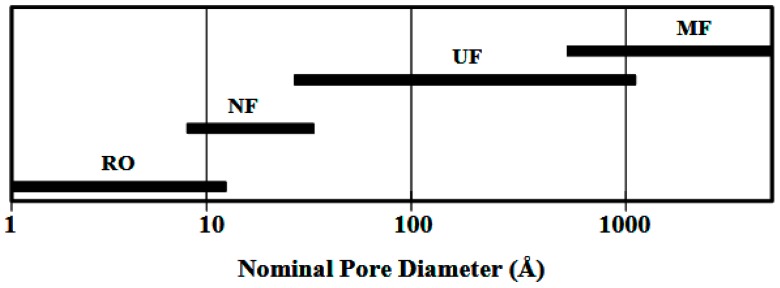
Range of nominal membrane pore sizes.

Figure 4 shows the range of nominal membrane pore sizes [13].

### 3.1. RO

In TFC RO hollow fiber membranes, an ultra-thin membrane is deposited on a microporous substrate. However, it is difficult to make RO HF membranes and so far the use of hollow fibers in RO has not been completely successful.

The hollow fiber modules that have been frequently used for RO have the selective skin on the outer side of the fiber, which creates concentration polarization and enhanced fouling, leading to the decrease in efficiency [14]. Veríssimo et al. [15] presented a new technique for the preparation of RO composite hollow fibers (only for low pressure), with the selective film on the bore side of the fiber. The technique introduced an inert liquid buffer between the aqueous m-phenylene diamine and the organic trimesoylchloride solution. Verissimo et al. [15] used polyetherimide (PEI) hollow fiber membranes with PA coated on the bore side for desalination, etc. By using the organic liquid as the buffer layer, an average water permeability of 0.6 × 10^−5^ Lm^−2^ h^−1^ Pa^−1^ and an NaCl rejection of 99.5% under a pressure of 10 × 10^5^ Pa (10 bar) feed pressure were observed, which is a very high rejection obtained with MPD/TMC based TFC-membranes. It was suggested that the membrane prepared using inert organic liquid was virtually defect free. By treating the membrane with formic acid, the average water permeability was improved (3 × 10^−5^ L m^−2^ h^−1^ Pa^−1^), with rejections higher than 95%.

Ni et al. [16] deposited PA layer via IP on a PSF UF hollow fiber membrane and treated it with NaOCl and polyvinly alcohol (PVA) solution. It was observed that the salt rejection of the membranes increased dramatically after the treatment with the NaOCl and PVA solution, but the permeate flux decreased slightly. A salt rejection of 96.3% and pure water flux of 10.9 L·m^−2^·h^−1^ were obtained when the membrane was treated with 500 ppm of NaOCl solution and 20 ppm of PVA solution.

Kim and Deng [17] modified the surface of commercial thin-film composite (TFC) membranes (six commercial TFC membranes–three nanofiltration (NF) (NF90, NF270, and DK) and three reverse osmosis (RO) membranes (XLE, BW30, and SG)) via low-pressure ammonia (NH_3_) plasma treatment. The contact angles of NH_3_ plasma treated membranes were decreased with increasing plasma treatment time. It was also revealed that modified membranes showed better characteristics in comparison with TFC membranes without modification for water treatment.

SRI’s (Stanford Research Institute) Materials Research Laboratory is developing an advanced hollow-fiber membrane for reverse osmosis based on a sulfonated-imidazole (SIM) polymer. It is currently being tested for shipboard desalination of seawater and brackish water. In addition to applications in desalination, membranes are also being explored for other types of water purification, such as to purify water extracted during gas and oil production. (SRI International, Menlo Park, CA, USA)

### 3.2. NF

**Figure 5:**
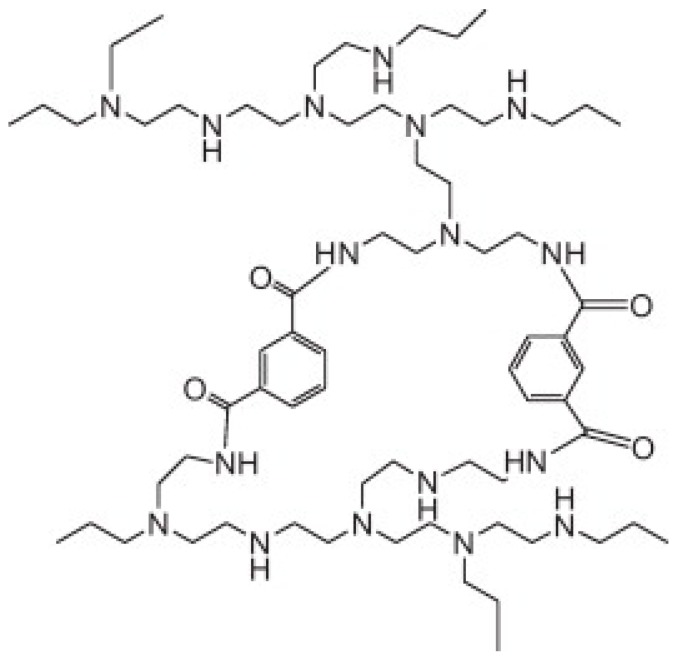
Possible chemical structure of the interfacially polymerized network formed with hyperbranched polyethyleneimine and isophthaloyl chloride [18].

Significant research work on TFC hollow fiber membranes for application in nanofiltration has been done. Sun et al. [18], for the first time, fabricated dual-layer thin-film composite nanofiltration hollow fibers (Torlon^®^ 4000T-MV polyamide-imide (PAI)) via IP of hyper branched polyethyleneimine (HPEI) and isophthaloyl chloride (IPC). The membrane provides minimal transport resistance and sufficient mechanical strength for water permeation under high pressures due to its negatively charged substrate and positively charged selective layer. The possible chemical structure of the interfacially polymerized thin film is proposed in Figure 5.

The newly developed NF membrane showed superior rejections (over 99%) for both positively and negatively charged dye molecules due to the steric-hindrance and the solute electro-neutrality effects (double-repulsion effect). The membrane showed a water permeability of 4.9 L m^−2^ bar^−1^ h^−1^ and an MWCO (molecular weight cut off) of 500 Da.

Liu et al. [19] fabricated a high flux thin film composite (TFC) hollow fiber nanofiltration (NF) membrane with a barrier layer of polypiperazine amide synthesized via interfacial polymerization (IP) on a previously prepared dual-layer (PES/PVDF) hollow fiber substrate. The novel hollow fiber NF membrane has a permeability of 16.6 L m^−2^ h^−1^ bar^−1^, an MWCO of 330 Da, and a tensile strength of 10.3 MPa.

Zheng et al. [20] prepared positively charged thin-film composite hollow fiber nanofiltration membranes by the dip-coating method using polypropylene hollow fiber microfiltration membrane as the support. The coating materials were polyvinyl alcohol (PVA) and polyquaternium-10 (the reaction product of hydroxyethyl cellulose with a trimethylammonium substituted epoxide, PQ-10), and glutaraldehyde was used as cross-linking agent. By using the submerged filtration technique, it was observed that the rejections of Brilliant Green, Victoria Blue B, and Crystal Violet were 99.8%, 99.8%, and 99.2%, respectively. It was found that the salt rejection order of the membrane was CaCl_2_ > MgCl_2_ > NaCl > MgSO_4_ > Na_2_SO_4_ at a neutral pH.

Maurya et al. [21] coated a thin polyamide layer on to PSF hollow fiber via IP (MPD and TMC) under different conditions, and used it for the separation of dyes (reactive black-5 and rhodamine-B) from water. Depending on the thickness of the PA-PSF membrane, the membranes exhibited about a 60–97% dye rejection and a product flux of 10–35 mL m^−2^ h^−1^.

Yu et al. [22] coated sodium carboxymethyl cellulose (CMCNa) on the outer surface of PP hollow fibers followed by cross-linking with FeCl_3_, and used it for the removal of anionic dyes (Congo red and Methyl blue) from aqueous solution. It was reported that the membrane’s MWCO was about 700 Da. The membrane could effectively remove anionic dyes (Congo red and Methyl blue) from aqueous solution.

Wei et al. [23] removed heavy metals from electroplating wastewater by using thin-film composite nanofiltration (NF) hollow-fiber membranes. TFC NF hollow-fiber membranes (PSF/PES) were prepared by IP technique using PIP and TMC as reactive monomers to form the PA on the inner surface of the hollow-fiber membrane. The membrane showed a pure water flux of approximately 152 L m^−2^ h^−1^ at 0.1 MPa. The rejection rates for chromium, copper, and nickel ions were 95.76%, 95.33%, and 94.99%, respectively.

Liu et al. [24] fabricated PA/PVDF hollow fiber composite NF membranes by IP on the external surface using TMC and PIP as the IP reaction reagents. It was observed that the rejections of PA/PVDF hollow fiber composite NF membranes for Na_2_SO_4_, MgCl_2_, KCl, NaCl, PEG600, and PEG1000 were 92.3%, 7.0%, 9.5%, 14.2%, 88.4%, and 89.3%, respectively.

Plisko et al. [25] developed TFN HF by incorporating fullerenol C_60_(OH)_22–24_ into the PA selective layer. The membranes were fabricated via the IP technique by alternately pumping fullerenol into the triethylenetetramine (TETA) aqueous solution and isophthaloyl chloride solution into the hexane through the PSF hollow fiber membranes. It was suggested by the authors that fullerenol may form hydrogen bonds with the amine and amide groups of the polyamide obtained by IP and ester bonds with unreacted acyl chloride groups. Antifouling properties of the membranes were evaluated by the flux changes during alternative filtration of pure water and foulant—0.4 g L^−1^ lysozyme solution in phosphate buffer (0.05 M, pH = 7.0). It was revealed that upon incorporation of fullerenol into the PA skin layer, the pure water flux decreased with a slight increase in the lysozyme rejection. It was reported that the properties of the modified membrane were superior to the unmodified membrane.

Abolfazli and Rahimpour [26] investigated the effect of adding silica nanoparticles and TETA (triethylenetetramine) on the permeation performance and morphology of thin-film composite hollow fiber membranes. In this study, PSF HF UF was used as a support layer, and the organic phase that contained TMC and the aqueous phase that contained piperazine, TETA, and SiO_2_ nanoparticles were used for IP reaction. The TFC skin layer was deposited on the external surface of the hollow fiber. Using a cross-flow hollow fiber filtration setup, membrane performances were investigated by using 2000 ppm of NaCl as feed at a pressure of 5 bar. It was observed that the salt rejection increased from 15.17% to 25.44% when the TETA concentration was increased from 0.5% to 10% (*w*/*v*). Further, it was also observed that the structure and performance of the composite membrane can be effectively controlled by adjusting the concentration of the additives.

Fang et al. [27] fabricated a composite hollow fiber, with a thin-film selective layer on the inner surface of a PES UF membrane as a substrate. The selective layer was formed by IP using branched PEI and TMC as the monomers in the aqueous and organic phases, respectively. The membrane prepared with the optimized preparation parameters showed a positively charged thin-film selective layer with a PWP of about 17 L m^−2^ h^−1^ bar and an MWCO of around 500 Da. It was observed that the membrane rejections for Mg^2+^ and Ca^2+^ ions were around 90% and the water flux was about 20 L m^−2^ h^−1^ at a 2 bar pressure. It was suggested by the authors that these membranes could be used in water softening applications.

### 3.3. FO

FO desalination utilizes osmotic pressure difference between a feed solution (FS) and a draw solution (DS) to drive water across a semi-permeable membrane. Higher rejections to a wide range of contaminants and low membrane fouling compared to the conventional RO process are drawing significant attention to the use of the FO process in water treatment.

**Figure 6:**
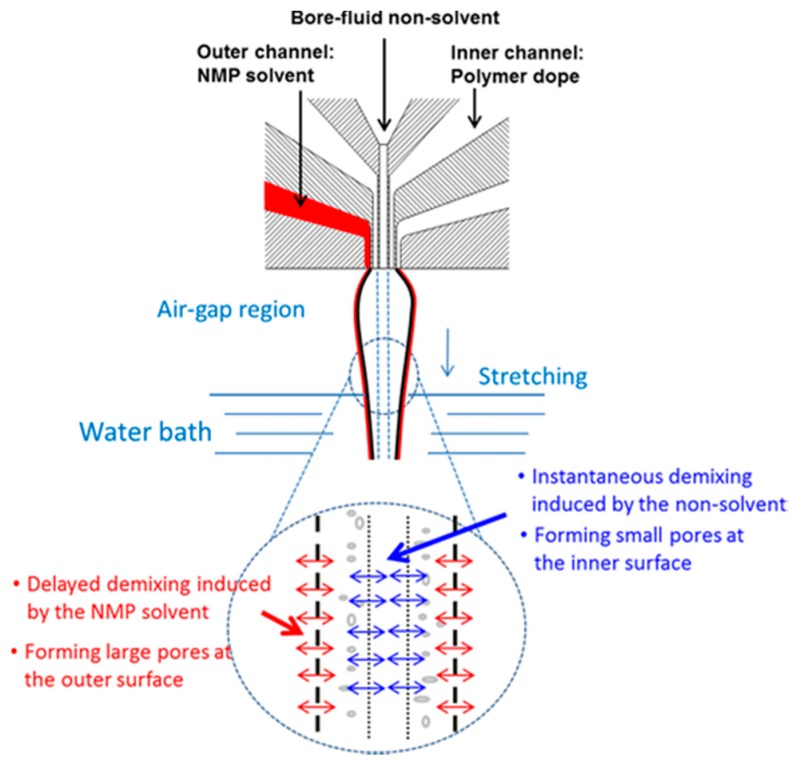
Strategies to control the phase inversion process with the aid of coextrusion technology employing a dual-layer spinneret [28].

Sukitpaneenit and Chung [28] presented TFC FO HFs for forward osmosis (FO) applications. A functional selective polyamide layer was formed by highly reproducible IP on a polyethersulfone (PES) hollow fiber support. PES HF support was made by dual-layer coextrusion technology to form desirable macrovoid-free and fully sponge-like morphology. Figure 6 shows strategies to control the phase inversion process with the aid of coextrusion technology employing a dual-layer spinneret.

The membrane exhibited relatively high water fluxes of 32–34 L m^−2^ h^−1^ and up to 57–65 L m^−2^ h^−1^ from pure water feed to 2 M NaCl draw solution tested under the FO and pressure retarded osmosis (PRO) modes, respectively. Authors claimed that the membranes could display a high water flux up to 15–18 L m^−2^ h^−1^ with seawater, which is comparable to the best value reported for seawater desalination applications.

Zhong et al. [29] discussed a new approach to fabricate thin-film composite (TFC) hollow fiber membranes for FO applications. A PA layer via IP was deposited on the lumen side of a hollow fiber, which was fabricated from sulfonated polyphenylenesulfone (PPSU). It was reported that sulfonating plays a role both in creating a macrovoid-free structure and inducing hydrophilicity to bring about higher water fluxes. It was revealed that the resultant membrane exhibited extremely high water fluxes of 30.6 and 82.0 L m^−2^ h^−1^ against a pure water feed using 2.0 M NaCl as the draw solution tested under FO and pressure retarded osmosis (PRO) modes, respectively, while maintaining low salt reverse fluxes below 12.7 g m^−2^ h^−1^.

**Figure 7:**
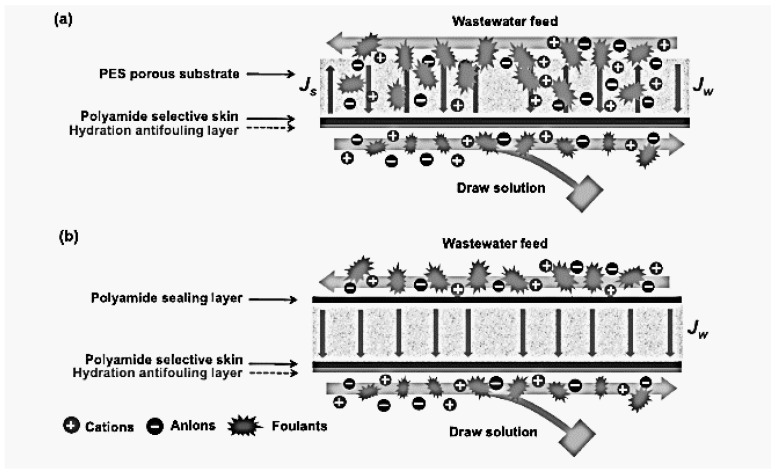
Schematic of fouling phenomena: (**a**) The conventional thin-film composite (TFC) membrane and (**b**) the newly developed double-skin TFC membrane in forward osmosis (FO) (under the pressure-retarded osmosis (PRO) mode) and PRO processes [30].

Gang et al. [30] deposited a PA skin onto the outer and inner surfaces of the PES fiber via IP between MPD and TMC, separately. FO and PRO tests were done to see its performance, with the draw solution flowing in the lumen side of the double skin TFC(dTFC)-PES membrane. Superior antifouling performance has been demonstrated by the dTFC-PES membrane when the feed was real wastewater brine (WWBr). The membrane water flux dropped slightly to 71% of the initial value at a high feed recovery of 80% in the FO mode. Stable performance was observed in the PRO mode where the power density only decreased to 90.8% of the initial value after a 12 h test at Δ*P* = 15 bar. Figure 7 shows the fouling mechanisms of conventional TFC FO membranes and the developed double-skin dTFC-PES membrane. Insignificant fouling was observed on the draw solution side. This phenomenon was due to the outstanding rejection of the polyamide skin facing the draw solution and the transmembrane water permeation from the feed side.

Chou et al. [31] deposited PA layer via IP (MPD-aqueous solution and TMC-hexane solution) on the inner surface of a PES hollow fiber and achieved a water flux of 12.4 L m^−2^ h^−1^ for 0.59 M NaCl feed solution and 2 M NaCl draw solution. It was suggested by the authors that this new FO hollow membrane has potential for seawater. Chou’s membrane also exhibited excellent intrinsic separation properties, with a water flux of 42.6 L m^−2^ h^−1^ using 0.5 M NaCl as the draw solution and DI water as the feed, with the active layer facing the draw solution orientation at 23 °C [32].

Chung’s group [6] designed and fabricated a triangle tri-bore spinneret for the fabrication of TFC HF membranes for FO processes. Using the IP technique, the PA selective layer was deposited on the inner surface of the Matrimid^®^ HF. The fabricated membrane showed an impressive mechanical strength with significant permeation properties. Their group also investigated the effects of glycerol treatment and surfactant concentration during the IP. The newly developed TFC tri-bore HF membranes exhibited high water fluxes of 50.5 and 11.8 L m^−2^ h^−1^, with salt leakages as low as 3.5 and 2.5 g m^−2^ h^−1^ in the PRO and FO mode, respectively, when using 2 M NaCl as the draw solution and pure water as the feed. In desalination experiments, the water flux was 5.8 L m^−2^ h^−1^ using a 2 M NaCl draw solution and a model seawater solution as the feed. Theoretically, triangle TFC tri-bore HF membranes have a higher water output per module than the round bore HF at the same packing density due to a larger effective membrane area.

Fang [33] fabricated thin film composite forward osmosis PES hollow fiber membranes by depositing a thin film active layer on the lumen side via IP. Two monomers, i.e., m-phenylenediamine (MPD) and TMC, were used for polymerization. From their studies, it was revealed that two different chemicals can successfully work together as the monomers in the aqueous solution to achieve a better performing membrane than applying each additive separately. Under optimized conditions, the water flux can reach 42.6 L h^−1^ m^−2^ using 0.5 M NaCl as the draw solution with the fabricated membrane. Thus, the performance of the fabricated FO hollow fiber was believed to be superior to all FO membranes reported in the literature.

Shibuya et al. [34] fabricated two types (different inner diameters) of TFC polyketone (as support) HFs with a PA layer on the shell side of the fiber. The PA layer was deposited via IP of TMC and MPD. The smaller diameter TFC hollow fiber showed better results than the larger diameter TFC HF in terms of a higher FO flux and better mechanical properties.

Ren and McCutcheon [35] used commercial ultrafiltration hollow fiber membranes (Koch Membrane Systems, Wilmington, MA, USA) as supports for polyamide TFC membranes and tested them for their FO performance. It was revealed the membranes exhibited remarkably good FO performance (both water and solute flux). It suggested that by using these commercial membranes as support material for making TFC, membranes do not need tailoring or adjustment.

### 3.4. PRO

PRO can be viewed as an intermediate process between FO and RO, where hydraulic pressure is applied in the opposite direction of the osmotic pressure gradient (similar to RO). Loeb and Norman [36] proposed a pressure-retarded osmosis (PRO) process. In a PRO process, water flows naturally from a low salinity stream (feed water) at an ambient pressure across a semi-permeable membrane to a pressurized high salinity stream (draw solution) driven by the osmotic pressure difference across the membrane. Chou et al. [37], for the first time, reported the fabrication of TFC HFMs, which could be used in the PRO process. A composite hollow fiber membrane was prepared by depositing a thin layer of PA on a PES hollow fiber via IP. The main reagents used for the IP were MPD, TMC, and cyclohexane. From the performance test, it was revealed that the TFC PRO hollow fiber membranes had a water permeability of 9.22 × 10^−12^ m s^−1^ Pa^−1^, salt permeability of 3.86 × 10^−8^ m s^−1^, and a structural parameter of 4.6 × 10^−4^ m.

Ingole et al. [38] developed a high flux and high power density TFC-PRO membrane from the PES membrane support, which was modified by coating with polydopamine (PDA), based on the easy self-polymerization and strong adhesion characteristics of dopamine (DA) under mild conditions. It was found that this improved process resulted in TFC-PRO membranes with simultaneous enhancements in both water permeability and salt rejection properties. On studying the performance of the TFC membrane with the PDA layer, it was revealed that the PDA-TFC membrane has a very high flux, excellent power density performance, and good stability. It was suggested by the authors that these membranes can be used in all engineered osmosis applications, including pressure retarded osmosis.

Wan et al. [39] developed an inner-selective thin-film composite (TFC) PRO HFM with a high operating pressure and a high power density by adding CaCl_2_, to the PES dope solution when the substrate HF was spun. As the dope solution viscosity increases due to the presence of CaCl_2_, the resultant membrane had a narrow pore size distribution and smaller pore sizes in comparison with HFs without CaCl_2_. Air was purged after circulating MPD aqueous solution containing 0.1 wt % SDS to make the surface dry. Next, hexane solution with 0.15 wt % TMC was circulated to react with MPD, resulting in absorption of the polymer on the inner surface of the hollow fiber substrate to form a thin PA selective layer. It was revealed that the TFC-PES membrane had the highest pure water permeability and smallest salt permeability. Further, it showed the highest ever reported power density of 38 W m^−2^ at 30 bar using 1.2 M NaCl solution and DI water as the draw and feed solutions, respectively.

Although the membrane technology is a well-established and is an advanced method for water treatment, it still needs to solve the problems that arise from membrane fouling and membrane chemical stability. Fouling mitigation will reduce the water production cost by extending the operational life time and lowering energy consumption.

Table 1 summarizes the TFC hollow fiber membranes for water treatment.

## 4. Pervaporation

### Alcohol Dehydration

The role of alcohols in our daily life is very common as they are widely used as solvents, cleaning agents, raw materials, or chemical intermediates for organic synthesis, industries, and the pharmaceutical and medical fields, etc. In the production and recycling of alcohols, dehydration of the alcohol/water mixture is a critical issue. An azeotropic mixture is formed by alcohol (ethanol, isopropanol, or butanol) and water, which makes purification of alcohols by conventional methods, such as distillation, inefficient and uneconomical. The pervaporation process for the purification of alcohols is energy saving, able to break azeotropes with a high separation efficiency, and is friendly to environment. Thus, industries are showing considerable interest in the pervaporation process for the dehydration/recycling of alcohol mixtures; mostly, it is the separation of the isopropanol-water mixture.

The novel organic-inorganic TFC membranes exhibit pervaporation separation performance surpassing most polymeric membranes and inorganic ceramic membranes previously developed for isopropanol dehydration [40].

Chung and his coworkers [40,41] reported the fabrication and characterization of TFC hollow fiber membranes for the pervaporation dehydration of isopropanol via proper monomer selection and optimal IP reaction.

Sukitpaneenit and Chung [42] fabricated a membrane with a thin selective PA layer, which was formed by IP, onto a porous PES HF support (free of macrovoids) fabricated via dual-layer co-extrusion technology. The surface of the TFC was modified by coating with either polydopamine or silicone rubber. The membrane was used for the separation of the ethanol/water mixture via pervaporation. Both coated membrane exhibited high water separation factors (polydopamine—51; silicone rubber—60) with high fluxes (polydopamine—6.6 kg m^−2^ h^−1^ and silicone rubber—607.5 kg m^−2^ h^−1^).

Zuo et al. [41] developed a novel TFC hollow fiber membrane comprising a PA selective layer on a porous polyamide-imide substrate. Four different amines (m-phenylenediamine (MPD) and three different molecular weight hyper-branched polyethyleneimine (HPEI)) were used for IP for the formation of the thin film on the outer surface of a hollow fiber. These membranes were used for the separation of an isopropyl alcohol (IPA)/water mixture via pervaporation, and it was reported that the TFC membrane fabricated from HPEI with a molecular weight of 2 kg mol^−1^ showed the best separation factor of 624 with a flux of 1282 g m^−2^ h^−1^. A permeate water concentration of 99.1 wt % was obtained with a feed composition of 85/15 wt % The IPA/water was at 50 °C under the optimal IP conditions.

In another article, Zuo et al. [40] described novel organic–inorganic TFC HF membranes with the introduction of an inorganic component, 3-glycidyloxypropyltrimethoxy-silane (GOTMS), in the chemical structure of the in situ synthesized polyamide layer. The performance testing of these membranes revealed that the separation factor surpassed most prior polymeric membranes and inorganic ceramic membranes for isopropanol dehydration. Flux data obtained from the organic–inorganic TFC HF membranes was 3.5 kg m^−2^ h^−1^ with a separation factor of 278 for a feed composition of 85/15 wt % isopropanol (IPA)/water at 50 °C.

Tsai et al. [43] fabricated a dual layer PA/PAN composite hollow fiber membrane via a fabrication technique involving simultaneous extrusion of two different dope solutions by using a triple orifice spinneret. SiO_2_ was added into the dope to increase the hydrophilicity of the dope. It was found that the PA layer adhered to the lumen surface of the PAN hollow fiber membrane. The membrane performance was studied for the separation of 90 wt % aqueous isopropanol solution at 25 °C by pervaporation. A permeation flux of 419 g m^−2^ h^−1^ and water content of 96.6 wt % in permeate was obtained.

Hua et al. [44] described the fabrication of thin-film composite tri-bore hollow fiber (TFC TbHF) membranes for the application of IPA dehydration. By conducting interfacial polymerization on the HF substrates, TFC HF membranes were prepared using the monomer, MPD, and another monomer, TMC. The membrane was tested for the dehydration of IPA. It was reported by the authors that the molecularly designed TFC TbHF membranes exhibited excellent pervaporation performance for IPA dehydration. The TFC TbHF membrane showed a flux of 2.65 kg m^−2^ h^−1^ with a separation factor of 246 for water/IPA separation at 50 °C using 85/15 wt % IPA/water as the feed.

In another publication, Hua et al. [45] described the Teflon AF2400/Ultem composite HF membrane preparation with good vapor permeation separation performance, including excellent thermal stability, for alcohol dehydration. The composite HF membranes were fabricated by dip coating a Teflon AF2400 layer on the outer surface of Ultem HFs. Developed composite HF showed a promising and stable separation performance with a flux of 4265 g m^−2^ h^−1^ and a separation factor of 383 for 95% isopropanol dehydration at 125 °C.

Table 2 summarizes TFC hollow fiber membranes for the separation and dehydration of alcohols via pervaporation.

## 5. Gas Separation

Thin film composite HF membranes for gas separations are usually prepared by coating the membrane surfaces via the dip coating technique. The other technique i.e., IP, is not successful or common.

### 5.1. Dip Coating Membranes

A porous HF, which cannot separate gases to a significant extent, can achieve its intrinsic separation properties by coating with an appropriate material [46]. Henis and Tripodi suggested that the separation properties of coated composite membranes are governed by the porous support material rather than by the coating material, and presented a resistance model to explain the behavior of composite gas separation membranes. Thus, Henis and Tripodi [47,48] developed composite membranes with an acceptable permeability and very high permselectivity [49,50].

The membrane consists of a porous asymmetric substrate of one polymer with a dense layer and a coating of another polymer—a second layer that is made by a non-selective and highly permeable material. On coating, there could be two possibilities:

i If the pores of the substrate membrane are very large, its gas flow resistance is very low. In this case, the thin layer coated on top of the substrate will govern both the flux and selectivity; or
ii in the other case, the substrate membrane is considered as a solution diffusion membrane with some defective pores. The substrate governs the membrane performance. The coated layer is only to stop the gas leakage by filling the defective pores.

It is difficult to know which mechanism is working. However, normally, PEBAX coating is thought to be in the first category and PDMS in the second category. However, it is controversial. There could be other influencing factors, such as the chemistry of the membrane, chemistry of the polymers used, and some other unknown factors.

### 5.2. IP Membranes

Few membranes were successfully made by this method for gas separation. Dip coating can also be applied when there are defects in the membrane prepared by IP.

### 5.3. CO_2_/Methane and CO_2_/Nitrogen Separation

Climate change and global warming is an environmental issue and it is caused mainly due to the rise in CO_2_ levels in the atmosphere. To lower the atmospheric CO_2_ concentration, carbon capture and storage (CCS) and the improvement of energy utility are important strategies. Flue gas refers to the combustion exhaust gas produced at power plants. Its composition depends on what is being burned, however, it usually consists of mostly nitrogen (typically more than two-thirds), carbon dioxide (CO_2_), and water vapor. Gas separation membranes have been commercialized for this purpose and are continuously growing [51].

A defect-free PDMS/PAN composite hollow fiber membrane with high performance for flue gas and air separations was fabricated by Liang et al. [52]. The dip coating process was used to make PDMS/PAN composite membranes. The selective layer (PDMS) was 230 nm thick on PAN HF. From the permeance test, it was found that the CO_2_ permeance was >5000 GPU and the CO_2_/N_2_ selectivity was about 11. The developed composite membrane could be used for various gas separations, such as the production of oxygen-enriched air and the separation of CO_2_ from flue gas. The membrane showed excellent O_2_ and CO_2_ permeances higher than 1000 and 5000 GPU, respectively, and the selectivity of O_2_/N_2_ and CO_2_/N_2_ were about 2 and 11, respectively.

Liu et al. [53] fabricated a membrane by coating a thin layer of poly(ether block amide (PEBA) onto microporous PEI hollow fiber substrate membranes. The membrane was prepared by coating a thin layer of PEBA onto microporous PEI hollow fiber substrate membranes. The membrane was tested for CO_2_/N_2_ separation and it was revealed that at 790 kPa a permeate stream containing 62 mol % CO_2_ was obtained, corresponding to 20% CO_2_ recovery. On the other hand, 99.4 mol % N_2_ can be obtained in the residue (nitrogen recovery 72%).

Fam et al. [54] prepared defect free Pebax^®^1657/[emim][BF_4_] gel membranes in the form of TFC hollow-fiber membranes. On investigation, TFC gel membranes showed excellent mechanical durability, including high packing density. The membrane was used to separate CO_2_ from a mixed-gas feed containing traces of water vapor and NO_x_. From the experimental results, the permeability of CO_2_ was very high, with CO_2_/N_2_ and CO_2_/CH_4_ selectivity of 36 and 15, respectively. It was suggested that the TFC gel membranes have the potential application for CO_2_ capture with a real gas feed.

Lasseuguette et al. [55] used four kinds of TFC hollow fibers membranes for gas separation by using two different porous supports (MicroPES and Oxyphan) and two different permeable polymers (PTMSP and Teflon AF2400) for coating. These membranes exhibited high CO_2_ and N_2_ permeability with CO_2_/N_2_ selectivity between 3.4 and 2.5.

Borisov et al. [56] modified polysulfone (PSF) hollow fiber membranes by air plasma and the Piranha etch (H_2_O_2_ + H_2_SO_4_) for the development of high efficiency support for composite membrane preparation. For composite membrane formation polymer solution (poly[1-(trimethylsilyl)-1-propyne], (PTMSP) in hexane) was forced in the HF’s lumen. The TFC HF fabricated on an air plasma treated surface showed a high CO_2_ permeance (3.3 × 10^5^ GPU) in comparison with unmodified membranes, including the highest surface energy (more phobic to hydrocarbons).

Zulhairun et al. [57] coated PDMS containing a Cu_3_(BTC)_2_ MOF layer onto a PSF hollow fiber and investigated its gas permeation properties. It was reported that the pure gas permeation experiment with CO_2_, N_2_, and CH_4_ corroborated the contribution of the Cu_3_(BTC)_2_ particle to the overall composite/hybrid membrane performance. The gas permeation rates were increased with an increasing number of PDMS–Cu_3_(BTC)_2_ coatings. CO_2_ permeance increased from 69.7 to 109.2 × 10^−6^ cm^3^ (STP)/cm^2^ s cmHg after five consecutive coatings. In addition, the CO_2_/CH_4_ and CO_2_/N_2_ selectivity were found to be increased as well. Cu_3_(BTC)_2_ contributed to a higher affinity toward CO_2_.

### 5.4. SO_2_ Removal

To capture CO_2_ from the combustion of fossil fuel plants, CO_2_ capture and separation (CCS) technologies, which are often based on the chemical absorption process, are used. The SO_2_ in the emission gas affects the sorbents in the CCS process. Flue gas from coal-power plants contains 6–196 ppm of SO_2_ after the CCS process. To remove the remaining SO_2_, another CCS process is necessary. Some studies have been made to apply polymeric membranes for flue gas applications.

Kim et al. [58] prepared a PEBAX/PEI hollow fiber composite membrane via coating poly(ether-b-amide) (PEBAX) onto a PEI hollow fiber and used it to remove SO_2_ from mixed gases. It was observed that the permeance of SO_2_ and CO_2_ increased with the operating pressure. However, the permeance of N_2_ was negligibly changed. With an increase in temperature, the permeation of SO_2_ sharply decreased. A similar phenomenon was also observed for the selectivity of SO_2_/CO_2_.

Kim et al. [59] prepared a hollow fiber composite membrane by coating PEI HF with a poly(vinyl chloride)-graft-poly(oxyethylenemethacrylate (PVC-g-POEM)). The inner and outer diameters of the HFM was 261 and 429 µm, respectively, and the selective coating layer on the outer surface was around 0.1µm. The membrane was tested for the permeance of pure gases (SO_2_, CO_2_, and N_2_) at different operating conditions. It was reported that the permeance of SO_2_ was 105–2705 GPU and the selectivity of SO_2_/CO_2_ was 3.9–175.6. From the mixed gas separation experiment, the maximum SO_2_ removal efficiency reached 84.5%.

### 5.5. O_2_/N_2_ Separation

Liang et al. [52] reported that the selectivity of O_2_/N_2_ was 2 and permeance of O_2_ was higher than 1000 GPU with a defect free PDMS/PAN composite hollow fiber membrane. Chong et al. [60] investigated the effects of different coating materials on the separation properties for the oxygen/nitrogen of PSF hollow fiber. The outer surfaces of two PSF hollow fiber were coated with PDMS and PEBAX (poly (ether block amide)) at different concentrations (1, 3, and 5 wt %). It was revealed that the permeance for oxygen gas was in the range of 38–73 GPU, while for nitrogen the range was 10–17 GPU. The uncoated membrane showed 62.35 and 15.11 GPU for the oxygen and nitrogen gas, respectively. Meng et al. [61] used PDMS coated PVDF hollow fiber membranes to study gas permeation (N_2_/O_2_). Data obtained experimentally from N_2_/O_2_ permeation supports the data theoretically obtained from the pore distribution model. Liang et al. [62] reported an N_2_ permeance of about 280 GPU, an O_2_/N_2_ selectivity of 2.2, and a water vapor permeance ranging from about 800 to 3700 GPU for a defect-free thin film composite PDMS/PAN hollow fiber membrane.

Liang et al. [63] fabricated, for the first time, using polymers of intrinsic microporosity (PIMs) for the development of high performance defect free hollow fiber composite membranes via dip coating, and opened a new avenue for future research. The membrane consists of three layers:

i The top selective layer was fabricated from the nucleophilic substitution copolymerization between PIM and beta-cyclodextrin (β-CD) (referred to as PIM-CD);
ii polydimethylsiloxane (PDMS) was used as to make a gutter layer; and
iii the material used for the substrate was polyacrylonitrile (PAN).

The development of a PIM composite hollow fiber was based on the idea to introduce a cross-linked PDMS gutter layer between the PIM-CD selective layer and PAN substrates. Thus, it could mitigate the detrimental solvent effects during the dip coating. In addition, PIM is allowed to adhere onto the gutter layer. Additionally, the gutter layer redistributes the gas transport across the membrane. It was reported that the O_2_ and CO_2_ permeances of the membrane were 69 and 483 GPU, respectively. The O_2_/N_2_ and CO_2_/N_2_ selectivity was 3.2 and 22.5, respectively. Further, the O_2_/N_2_ and CO_2_/N_2_ selectivity was increased to 4.2 and 29.5, respectively, in air separation and flue gas tests.

Pian et al. [64] used a PDMS coated ceramic hollow fiber for oxygen enrichment from air, and achieved an oxygen permeance of 104 GPU with an O_2_/N_2_ ideal selectivity of 2.0. It was suggested that a ceramic hollow fiber-supported PDMS composite membrane could be a competitive oxygen enrichment membrane for industrial application.

### 5.6. Water Vapor Transport

For various applications, such as the dehydration of natural gas, air conditioning, storage of foodstuffs, aviation, and spaceflights, the removal of water vapor from the gas stream is necessary. Condensation and adsorption, which are conventional methods, are uneconomical. On the other hand, membrane technology is better, cheaper, and friendly to the environment.

Ingole et al. [65] fabricated a hydrophilic PA layer on the top of a polydopamine (PDA) coated PES hollow fiber membrane. The hydrophilic PA layer was prepared via IP using 3,5-diaminobenzoic acid (3,5-DABA) as an aqueous phase monomer and trimesoyl chloride (TMC) as an organic phase monomer. The membrane was studied for mixed vapor/gas (water vapor/N_2_ mixtures) separation. It was reported that the newly prepared TFC hollow fiber membranes showed a reasonably high selectivity (up to 195) and superior permeation fluxes (up to 3185 GPU).

In another work, Ingole et al. [66] studied the performance of the metal organic framework (MOF) incorporated thin film nanocomposite (TFN) membrane for water vapor transport from the gas mixture, and reported that it drastically enhanced the water vapor transport. The novel membrane is fabricated by depositing a nanocomposite layer of PA (IP of MPD and TMC) with the incorporation of MOF particles. The MOF particles NH_2_-MIL-125 (Ti) (amine-functionalized titanium metal organic framework) were prepared from Ti(BuO)_4_ (Titanium butoxide) using reflux and solvothermal reactions [67]. The presence of 0.1wt % MOF nanoparticles increased the permeance from 785 GPU (TFC without MOF) to 2244 GPU (MOF@TFN3) and the selectivity from 116 to 542.

Baig et al. [68] studied water vapor removal using a TFN hollow fiber polyamide-PSF membrane, decorating the surface with carboxylated TiO_2_ nanoparticles. Surfaces of pure TiO_2_ were modified by introducing functional groups to increase the hydrophilicity. The presence of modified TiO_2_ nanoparticles on the membrane surface increased the hydrophilicity of the TFN membrane due to excess carboxylic groups. From the water vapor permeation tests, it was revealed that the water vapor permeance and selectivity drastically increased in comparison with the data obtained for an unmodified membrane. At optimum conditions, the water vapor permeance and selectivity of 1340 GPU and 486, respectively, were obtained.

To produce a covalent organic polymers layer on the surface of a PES hollow fiber, four different aqueous phase monomers, i.e., 1,3-benzenedithiol (BDT), m-phenylenediamine (MPD), 1,3,5-benzenetrithol (BTT), and piperazine (PIP), along with trimesoyl chloride (TMC) for IP, were used by Ingole et al. [69]. Mixed gas, water vapor, and N_2_ were used to investigate the gas permeation activity of these TFC membranes. A superior result was exhibited by the TFC membrane with BDT for the water vapor permeance (2054 GPU) and the water vapor/N_2_ selectivity (119).

Yun et al. [70] deposited a PA layer via IP using 3,5-diaminobenzoic acid (BA) and TMC as the aqueous and organic phase monomers, respectively, on PEs hollow fiber. From the water vapor/nitrogen separation study, it was reported that the best permanence and separation factors were 2160 GPU and 23, respectively. It suggested that the performance of the TFC membrane depends on the chemistry of the selective layer.

Ingole et al. [71] synthesized a novel thin film nanocomposite (TFN) membrane hybridized with amino functionalized acid-activated bentonite (ABn-NH) clay. The membrane was characterized using physicochemical techniques, including X-ray diffraction, BET surface analysis, thermal gravimetric analysis (TGA), Fourier transform infrared (FTIR) spectroscopy, scanning electron microscopy (SEM), atomic force microscopy (AFM), and contact angle analysis. The concentration effect of ABn-NH clay (0–1.0 wt %) on the permeation of water vapor and N_2_ was investigated. It was observed that incorporation of ABn-NH particles into the polyamide membrane increased the permeance enormously. The best performance of the ABn-NH-TFN-3 membrane (vapor permeance of 2809 GPU and water vapor/N_2_ selectivity of 913) was achieved when additive loading was 0.5 wt %.

Table 3 shows the performance of TFC hollow fiber membranes for the separation of gas or gas/water vapor mixtures.

## 6. Summary

Nowadays, asymmetric thin film composite (TFC) polymeric hollow fiber (HF) membranes are extensively used in industrial gas/vapor separations and water treatment. Many attempts have been made during the past decade to develop high flux TFC HFs, however, the following trends are most visible:

(1) Among various methods to coat the thin selective layer on the porous sublayer, dip coating and IP are the most popular for practical applications;
(2) the thin selective layer is developed mostly on the lumen side of the HF;
(3) as for gas/vapor separation, coating of the thin surface layer is done mostly by dip coating. There are only a few examples of IP;
(4) as for water treatment, applications of TFC HFs in FO and PRO are newly investigated for desalination purposes;
(5) in pervaporation, applications of TFC HFs are most encouraging for alcohol dehydration; and
(6) high-performance multiple-layer PIM composite hollow fiber membranes for gas separation open a new avenue for researchers.

Very little work has been done so far for the development of ceramic TFC HFs. At present, TFC HFs are still in the early stage and need more research and development, especially to find the most desirable fabrication conditions. As the membrane technologies are economical, environmentally friendly, and easy to use, they are the leading choice for water purification, gas separation, dehydration of alcohols, separation of liquids, etc. They will continue to be so for many years to come. TFC HF membranes will play a major role for the future advancement of membrane technology.

## Figures and Tables

**Table 1:** TFC hollow fiber membrane for water treatment.

TFC HF Membrane	Use	Results	Ref.
Reverse Osmosis			
PDA-PES	Desalination	High flux, excellent power density performance, and good stability. Can be used in all engineered osmosis applications, including PRO.	[15]
PA-PSF, post treated with NaOCl And PVA solution	Desalination	Salt rejection of 96.3% and pure water flux of 10.9 L·m^−2^·h^−1^.	[16]
Low-pressure NH_3_ plasma treatment (six commercial TFC membranes (3 NF, 3 RO)	Water treatment	CA of NH_3_ plasma treated membranes were decreased with increasing plasma treatment time. Surface hydrophilicity increased.	[17]
Nano filtration			
Polypiperazine amide-PES/PVDF (NF)	Waste water treatment	High-flux and outstanding selectivity of TOC/TDS.	[19]
HPEI-Torlon^®^ PAI (NF)	Removal of organic matters from water	Water permeability 4.9 L m^−2^ bar^−1^ h^−1^.	[18]
PVA + PQ-10–PP (NF)	Desalination	Salt rejection order CaCl_2_ > MgCl_2_ > NaCl > MgSO_4_ > Na_2_SO_4_. Cationic dye removal (submerged filtration).	[20]
PA-PSF (NF)	Removal of dye etc. Water treatment	MWCO 490 to 730 g/mole. Rejections: Reactive black-5 and rhodamine-B 60–97%, water flux of 10–35 mL m^−2^ h^−1^ at 25 psi.	[21]
CMCNa/PP (NF)	Water treatment	MWCO 700 Da. Remove anionic dyes. The dye retention 99.8%, water permeability 7.0 L m^−2^ h^−1^ bar, salt rejection 99.8%.	[22]
PIP + TMC-PSF + PES (NF)	Heavy metal removal	Pure water flux of approximately 152 L m^−2^ h^−1^ at 0.1 MPa. Rejection rates for chromium, copper, and nickel ions were 95.76%, 95.33%, and 94.99%, respectively.	[23]
PA (TMC + PIP)-PVDF	Desalination	Rejections Na_2_SO_4_, MgCl_2_, KCl, NaCl, PEG600 and PEG1000—92.3%, 7.0%, 9.5%, 14.2%, 88.4%, and 89.3% respectively.	[24]
Si NPTs + TETA-PSF	Desalination	Rejection increased from 15.17% to 25.44% with increasing the TETA concentration from 0.5% to 10% (*w*/*v*). Concentration of additives affects the structure and performance.	[26]
Fullerene C_60_(OH)_22–24_ + PA-PSF (UF)	Water treatment	Superior antifouling properties. Decrease of pure water flux and a slight increase of rejection of lysozyme and PVP K-15. Superior antifouling properties. Correlation between surface properties and fouling behavior.	[25]
PA-PEI		PWP about 17 L m^−2^ h^−1^ bar^−1^. Rejections for Mg^2+^ and Ca^2+^ ions around 90%. Water flux 20 L m^−2^ h^−1^ at 2 bar pressure. Suitable for water softening applications.	[27]
Forward Osmosis, Pressure Retarded Osmosis			
Functional selective PA layer-PES (FO, PRO)	Desalination application	Relatively high water fluxes FO 32–34 L m^−2^ h^−1^. PRO 57–65 L m^−2^ h^−1^ (PRO) for pure water feed and 2 M NaCl as the draw solution. Model seawater solution as the feed, water flux up to 15–18 L m^−2^ h^−1^.	[28]
PA-PPSU	Desalination in FO process	FO and PRO. Higher water flux.	[29]
PA-PES (dTFC-PES) (FO, PRO)	Waste water treatment	High feed recovery of 80% in the FO mode. Stable performance was observed in the PRO mode.	[30]
PA-PES (FO)	Water treatment, desalination	Water flux 12.4 L m^−2^ h^−1^ for 3.5 wt % NaCl feed solution and 2 M NaCl draw solution.	[31]
PA-PES (FO)	Water treatment, desalination	Excellent intrinsic separation properties. Water flux of 42.6 L m^−2^ h^−1^ using 0.5 M NaCl as the draw solution.	[32]
PA-Matrimid^®^ (Tri-bore-composite) (PRO, FO)	Saline water treatment	High water fluxes of 50.5 L m^−2^ h^−1^ and 11.8 L m^−2^ h^−1^ with salt leakages as low as 3.5 and 2.5 g m^−2^ h^−1^, in PRO and FO modes, when using 2 M NaCl as the draw solution and pure water as the feed.	[6]
PA-polyketone (IP shell side)	Saline water treatment	HF with smaller diameter -higher FO flux and better mechanical properties than those larger diameter HF.	[34]
PA-PES	Desalination	Under optimized conditions, water flux—42.6 L h^−1^ m^−2^ using 0.5 M NaCl as draw solution. Superior performance as FO HF.	[33]
PA-PES (PRO)	Water treatment	Could be used in PRO process. Water permeability—9.22 × 10^−12^ ms^−1^ Pa^−1^. Salt permeability—3.86 × 10^−8^ m s^−1^. Structural parameter—4.6 × 10^−4^ m.	[37]
PDA-PES (PRO)	Water treatment	Very high flux, excellent power density performance, and good stability. Can be used in all engineered osmosis applications, including PRO.	[38]
PA-CaCl_2_ + PES	Saline water treatment	Highest pure water permeability. Very low salt permeability.	[39]

**Table 2:** TFC hollow fiber membrane for separation and dehydration of alcohols via pervaporation.

Membrane	Use	Result	Ref
Pervaporation			
PA (4 different MPD and 3 different HPEI)-PA + imide	Isopropanol dehydration	Separation factor of 624, flux 1282 g m^−2^ h^−1^, permeate 99.1 wt % water.	[41]
GOTMS + PA-Poly ether imide (Ultem^®^)	Isopropanol dehydration	Separation performance surpassing ceramic membranes.	[40]
PA-PES (dual layer), Surface modified polydopamine or silicone rubber	Ethanol dehydration	Water separation factors 51 and 60, high fluxes 6.6 and 7.5 kg m^−2^ h^−1^. Good selectivity/separation factors.	[42]
PA-PAN + SiO_2_ (dual layer, triple orifice spinneret)	Separation of 90 wt.% aqueous isopropanol solution	419 g m^−2^ h^−1^ of permeation flux and 96.6 wt % of water content in permeate.	[43]
Poly (ether imide) Ultem^®^ 1010 (tri-bore hollow fiber)	Isopropanol dehydration	Flux 2.65 kg m^−2^ h^−1^ with a separation factor of 246 for water/IPA separation at 50 °C using 85/15 wt % IPA/water as the feed.	[44]
Teflon AF2400 layer on the outer surface of Ultem HFs (dip coating)	Isopropanol dehydration	Flux 4265 g m^−2^ h^−1^, separation factor 383 for 95% isopropanol dehydration at 125 °C.	[45]

**Table 3:** TFC hollow fiber membrane for the separation of gas or gas/water vapor mixtures.

Membrane	Use	Result	Ref.
CO_2_/methane and CO_2_/nitrogen separation			
PEBA-PEI	CO_2_/N_2_	Permeate stream containing 62 mol % CO_2_ was obtained at a CO_2_ recovery of 20%. 99.4 mol %. N_2_ in the residue with a nitrogen recovery of 36%.	[54]
Four kinds of TFC composite HFM	Gas separation	High CO_2_ and N_2_ permeability. CO_2_/N_2_ selectivity’s 3.4–2.5.	[55]
Pebax^®^1657/[emim][BF_4_] gel membranes in the form of thin film composite hollow fiber membranes	Separation of CO_2_ mixed-gas containing traces of water vapor and NO_x_	Excellent mechanical durability, potential application for CO_2_ capture with real gas feed.	[54]
Modified air plasma and the Piranha etch PTMSP-PSF	CO_2_ permeance	High permeance (3.3 × 10^5^ GPU) in comparison with unmodified membranes including highest surface energy.	[56]
PDMS containing Cu_3_(BTC)_2_ MOF-PSF	Gas permeation properties	CO_2_ permeance increased from 69.7 to 109.2 × 10^−6^ cm^3^ (STP)/cm^2^ s cmHg. CO_2_/CH_4_ and CO_2_/N_2_ selectivity increased.	[57]
SO_2_ Removal			
PEBAX/PEI	Removal SO_2_ from mixed gases	Permeance of SO_2_ and CO_2_ increased.	[58]
PVC-g-POEM-PEI	Separation of gases. (SO_2_, CO_2_, N_2_)	Permeation of SO_2_ 105–2705 GPU, Selectivity SO_2_/CO_2_—3.9–175.6. Mixed gas separation SO_2_ removal efficiency reached up to 84.5%.	[59]
O_2_/N_2_ separation			
PDMS or PEBAX-PSF (DCo)	Oxygen enrichment	Higher permeance O_2_ and N_2_.	[60]
PDMS-PAN (DCo)	Gas separation	N_2_ permeance 280 GPU, O_2_/N_2_ selectivity of 2.2, water vapor permeance 800 to 3700 GPU.	[62]
PVDMS-PVDF (DCo)	Gas separation (N_2_/O_2_ separation)	Permeability and selectivity were in good agreement with the theoretical results.	[61]
PDMS-ceramic (DCo)	Oxygen enrichment from air	Permeance of 104 GPU with O_2_/N_2_ ideal selectivity of 2.0.	[64]
PIM-CD/PDMS/PAN	Gas separation O_2_, N_2_ and CO_2_	O_2_/N_2_ and CO_2_/N_2_ selectivity, 3.2 and 22.5, respectively.	[63]
Water vapor transport			
PDA-PES (DCo)	Water vapor/N_2_ mixtures	Excellent selectivity. Permeance 3185 GPU, selectivity 195.	[65]
MOF incorporated TFN NH_2_-MIL-125 (Ti)-PSF	Water vapor separation from flue gas	Water vapor permeance increased from 785 GPU (TFC) to 2244 GPU (MOF@TFN3). Selectivity 116 to 542 with NH_2_-MIL-125 (Ti) MOF.	[66]
ABn-NH-TFN-PSf	Water vapor/N_2_	Vapor permeance 2809 GPU, vapor/N_2_ selectivity 913.	[69]
Carboxylated TiO_2_ + PA-HF	Flue gas dehydration	Permeance 1340 GPU, selectivity 486.	[68]
BDT, MPD, BTT), PIP-PES (4 monomers used for IP)	Water vapor/N_2_	BDT exhibited superior results, water vapor permeance 2054 GPU and the water vapor/N_2_ selectivity 119.	[71]

## Abbreviations

ABn-NH	functionalized acid-activated bentonite clay
BDT	1,3 benzenedithiol
BTT	1,3,5-benzenetrithol
CMCNa	sodium carboxymethyl cellulose
3,5-DABA	3,5-diaminobenzoic acid
DCo	Dip coating
DI	deionized
DMS	Dimethylsiloxane
FS	functional selective
GOTMS	3-glycidyloxypropyltrimethoxy-silane
HFM	Hollow fiber membrane
HPEI	hyper branched polyethyleneimine
IP	interfacial polymerization
IPC	isophthaloyl chloride
MPD	m-phenylenediamine
MWCO	molecular weight cut off
NF	Nanofiltration
NPTs	Nanoparticles
PAN	Polyacrylonitrile
PAI	polyamide-imide
PDA	Polydopamine
PDMS	Polydimethylsiloxane
PEBAX	poly(ether block amide)
PES	Polyethersulfone
PIMs	polymers of intrinsic microporosity
PIP	Piperazine
PK	Polyketone
PP	Polypropylene
PPSU	Polyphenylenesulfone
PQ-10	polyquaternium-10
PRO	Pressure retarded osmosis
PSF	Polysulfone
PTMSP	poly[1-(trimethylsilyl)-1-propyne
PVA	polyvinyl alcohol
PVDF	Polyvinylene fluoride
PVP	poly (4-vinyl pyridine)
PWF	pure water flux
RO	reverse osmosis
SIR	Silicon rubber
TDS	total dissolved solids
TEOS	tetraethylorthosilicate
TEPA	Tetraethylenepentamine)
TETA	Triethylenetetramine
TFC	thin film composite
TFM	thin film membrane
TFN	thin film nanocomposite
Ti(OBu)_4_	Titanium butoxide
TMC	trimesoyl chloride
TOC	total organic carbon
